# Diagnostic Accuracy of American College of Radiology Ovarian-Adnexal Reporting and Data System (ACR O-RADS) Ultrasound for Characterizing Adnexal-Ovarian Masses: A Histopathological Correlation Study

**DOI:** 10.7759/cureus.117791

**Published:** 2026-10-12

**Authors:** Rupanshi Jain, Saurav Bhagat, B Sampath Akhila, Sakshi Aggarwal, Sarthak Jain, Urvi Jangir, Sarthak Jain

**Affiliations:** 1 Department of Radiology, Sharda School of Medical Sciences and Research, Sharda University, Greater Noida, IND; 2 Department of Obstetrics and Gynaecology, Shri Siddhi Vinayak Medical College and Hospital, Sambhal, IND; 3 Department of Pathology, Lady Hardinge Medical College, New Delhi, IND; 4 Department of Radiology, Amrita School of Medical Sciences and Research, Amrita Hospital, Faridabad, IND

**Keywords:** acr o-rads, adnexal masses, histopathology, malignancy risk, transvaginal ultrasound

## Abstract

Background

Accurate preoperative characterization of adnexal-ovarian masses is essential for appropriate clinical management. The American College of Radiology Ovarian-Adnexal Reporting and Data System (ACR O-RADS) ultrasound provides a standardized ultrasound-based framework for malignancy risk stratification.

Objective

The objective of this study is to evaluate the diagnostic accuracy of transvaginal ultrasonography (TVUS) using ACR O-RADS for adnexal-ovarian masses, with histopathological diagnosis as the reference standard.

Materials and methods

This prospective, single-center, hospital-based observational diagnostic-accuracy study with a cross-sectional index-test/reference-standard comparison was conducted over 18 months, from May 2023 to November 2024. Women with suspected adnexal-ovarian masses underwent TVUS, and each lesion was categorized according to the ACR O-RADS ultrasound system. Histopathological examination following surgical excision served as the reference standard. Sensitivity, specificity, positive predictive value (PPV), and negative predictive value (NPV) were calculated and reported with Wilson 95% CIs.

Results

Among the 34 adnexal-ovarian masses, histopathological examination classified 16/34 (47.1%; 95% CI: 31.5%-63.3%) as malignant, 15/34 (44.1%; 95% CI: 28.9%-60.5%) as benign, and 3/34 (8.8%; 95% CI: 3.0%-23.0%) as borderline. When O-RADS categories 4 and 5 were considered test-positive and malignant/borderline lesions were combined as disease-positive, O-RADS demonstrated a sensitivity of 100.0% (95% CI: 83.2%-100.0%), specificity of 66.7% (95% CI: 41.7%-84.8%), PPV of 79.2% (95% CI: 59.5%-90.8%), and NPV of 100.0% (95% CI: 72.2%-100.0%). O-RADS category was significantly associated with histopathological classification (p < 0.001); however, the proportion of malignant lesions was identical in O-RADS 4 and O-RADS 5 (66.7% each).

Conclusion

ACR O-RADS demonstrated promising diagnostic performance for characterizing adnexal-ovarian masses in this cohort. These findings require confirmation in larger, consecutively recruited populations before broader conclusions regarding clinical utility can be made.

## Introduction

Adnexal masses are frequent in gynecological practice and consist of a heterogeneous group of benign, borderline, and malignant lesions that originate from the ovary, fallopian tubes, or the surrounding pelvic structures [1]. Their clinical importance lies in their ability to be monitored non-surgically yet to be distinguishable from lesions where surgical intervention and/or gynecologic oncology referral would be indicated. Previously, the serum cancer antigen 125, menopausal status, and ultrasonographic features were used together to assess malignancy risk [1]. This distinction remains clinically important because ovarian cancer continues to contribute substantially to the global cancer burden [2]. The imaging modality of choice for the initial investigation of suspected adnexal masses is transvaginal ultrasonography, which is easily available and in practice can show the morphology and vascularization of the mass [3]. The International Ovarian Tumor Analysis group has provided standardized sonographic terminology that enhances the description of features like lesion composition, septations, papillary projections, solid components, acoustic shadows, and Doppler flow [3]. Malignant prediction was then enhanced by rule-based models, but this may still depend on the experience of the examiner, complexity of the lesion, and the uniform application of predefined descriptors [4].

Adnexal-ovarian lesions were categorized according to the American College of Radiology Ovarian-Adnexal Reporting and Data System ultrasound framework (ACR O-RADS US 2020) [5]. The system is based on the morphologic and Doppler vascularity of the lesions, making it possible for the radiologist and gynecologist to communicate risk in an organized way. There are studies that assess the related O-RADS approaches that have shown clinically useful diagnostic performance in ultrasound settings [6]. There are also magnetic resonance imaging (MRI) studies that have confirmed structured O-RADS assessment [7]. Recently, it has been demonstrated that the O-RADS ultrasound classifications can help distinguish benign from malignant adnexal masses, and that they may provide enhanced discriminatory power when used together with other biomarkers, like human epididymis protein 4 [8].

There has been an increasing body of evidence on the performance of O-RADS in various settings and over various populations. Pediatric investigations have reported a high degree of diagnostic agreement between reviewers, but disease prevalence and characteristics of lesions vary from those reported in adults [9]. There has been support for the utility of O-RADS for malignancy-risk stratification from large validation studies with greater than 1000 adnexal masses [10]. Its diagnostic utility in clinical routine has been studied in further detail in more recent multicenter studies, and this is still subject to external validation in different institutions and operator groups [11]. Additionally, novel artificial intelligence models are being evaluated against expert O-RADS evaluation, suggesting the potential for future use of explainable computational tools in the characterization of ovarian masses [12].

Objective of the study

This study aimed to evaluate the diagnostic accuracy of ACR O-RADS ultrasound for characterizing adnexal-ovarian masses using transvaginal ultrasonography, with histopathological diagnosis as the reference standard. Sensitivity, specificity, positive predictive value (PPV), and negative predictive value (NPV) were assessed as the principal diagnostic-performance measures.

## Materials and methods

Study design and setting

This prospective, single-center, hospital-based observational diagnostic-accuracy study used a cross-sectional index-test/reference-standard design and was conducted in the Department of Radiodiagnosis, Sharda Hospital, Greater Noida, Uttar Pradesh, over 18 months from May 2023 to November 2024. Consecutive adult women with clinical suspicion of an adnexal mass who fulfilled the eligibility criteria were recruited. Participants underwent targeted clinical assessment followed by transvaginal ultrasonography, and the ultrasound findings were subsequently compared with histopathological examination following surgery.

Study population

The study included women with clinically and/or sonographically suspected adnexal-ovarian masses who were referred for transvaginal ultrasonography. Women with pelvic masses (excluding ovarian) and pregnant women were excluded. Bilateral lesions were present in the source records; however, the thesis did not explicitly describe a prespecified rule for whether multiple lesions in the same patient were analyzed separately or represented by a single index lesion. This was therefore acknowledged as a methodological limitation.

Sample size

Consecutive adult women with suspected adnexal masses who fulfilled the eligibility criteria were recruited during the study period. The planned sample size was calculated using the single-proportion formula $n = \frac{Z^{2}P(1 - P)}{L^{2}}$, assuming a regional prevalence of adnexal masses of 7%, a 95% confidence level (Z = 1.96), and an absolute precision of approximately 8%, yielding a minimum planned sample size of 31 participants. The final analysis included 34 adnexal-ovarian masses with histopathological confirmation.

Transvaginal ultrasonography

Transvaginal ultrasonography was performed using a Philips EPIQ 7G grayscale real-time ultrasound system (Philips Ultrasound, Bothell, WA) in the Department of Radiodiagnosis. Each adnexal-ovarian lesion was systematically assessed for morphology, composition, locularity, margins, septations, papillary projections, solid components, calcifications, and Doppler vascularity. The source study records did not document the transvaginal probe specifications, number of ultrasound readers, individual reader experience, or a predefined procedure for resolving disagreements between readers; therefore, these details could not be reliably reported and were acknowledged as methodological limitations.

O-RADS classification

Adnexal-ovarian lesions were categorized according to the ACR O-RADS US 2020. Classification was based on lesion morphology, composition, locularity, wall and margin characteristics, papillary projections, solid components, and Doppler vascularity. The original study dataset additionally recorded Doppler vascularity on a 0-5 scale. Because this differs from the standardized ACR O-RADS color-score terminology and the source records did not provide definitions for each 0-5 score, these values were not interpreted as the standardized ACR O-RADS color score; instead, they were reported separately as study-recorded Doppler vascularity scores.

Histopathological examination

Surgically excised specimens were subjected to histopathological examination in the Department of Pathology. The final pathological diagnosis was established on the basis of routine histomorphological examination, and lesions were categorized as benign, borderline, or malignant. The final histopathological diagnosis served as the reference standard for comparison with the ACR O-RADS ultrasound classification. The original study records did not specify formal blinding procedures between ultrasound interpretation and histopathological assessment. The source records did not document formal blinding of the ultrasound reader to histopathological findings or of the pathologist to the ultrasound/O-RADS findings.

Study outcomes

The primary outcome was the diagnostic accuracy of O-RADS ultrasound classification for distinguishing malignant/borderline adnexal-ovarian lesions from benign lesions, using histopathological examination as the reference standard. The sensitivity, specificity, PPV, and NPV were used to evaluate the diagnostic performance. The association of individual ultrasonographic features and O-RADS categories with histopathological findings was also assessed.

Statistical analysis

Data were entered into Microsoft Excel (Microsoft® Corp., Redmond, WA) and analyzed using SPSS version 22.0 (IBM Corp., Armonk, NY). Categorical variables were summarized as frequencies and percentages, and continuous variables as mean ± standard deviation, as appropriate. Because several contingency tables contained small expected frequencies and zero cells, exact methods were used for categorical comparisons. Fisher’s exact test was applied to 2 × 2 tables, whereas the Fisher-Freeman-Halton exact test was used for larger contingency tables; Monte Carlo estimation was used when direct exact computation was not feasible. Cramér’s V was reported as the effect-size measure for categorical associations. Histopathological proportions were reported with 95% confidence intervals (CIs) calculated using the Wilson score method. For both the primary diagnostic-accuracy and receiver operating characteristic (ROC) analyses, malignant and borderline lesions were predefined as disease-positive, whereas benign lesions were classified as disease-negative. For the primary diagnostic-accuracy analysis, O-RADS categories 4 and 5 were considered test-positive and O-RADS category 3 test-negative.

Sensitivity, specificity, PPV, and NPV were calculated from the resulting 2 × 2 contingency table using histopathological examination as the reference standard, with corresponding 95% CIs calculated using the Wilson score method. For ROC analysis, the ordinal O-RADS category was used as the test variable, and the area under the ROC curve (AUC) was reported with its 95% CI. Monte Carlo estimation was used when direct exact computation was not feasible. Cramér’s V was reported as an effect-size measure for categorical associations. All statistical tests were two-sided, and p < 0.05 was considered statistically significant.

Ethical considerations

The study protocol was reviewed and approved by the Institutional Ethics Committee of the School of Medical Sciences and Research and Sharda Hospital, Sharda University, at its meeting held on March 28, 2023. The formal approval certificate (Ref. No. SU/SMS&R/76-A/2023/112) was issued on May 2, 2023. Thus, March 28, 2023, represents the date of committee approval, whereas May 2, 2023, represents the date on which the approval certificate was issued.

## Results

Histopathological distribution

Surgically excised specimens were subjected to histopathological examination, which served as the reference standard. Final diagnoses were categorized as benign, borderline, or malignant and subsequently compared with the preoperative O-RADS ultrasound classification. A total of 34 adnexal-ovarian masses were analyzed. Histopathological examination identified 16 lesions as malignant (47.1%; 95% CI: 31.5%-63.3%), 15 as benign (44.1%; 95% CI: 28.9%-60.5%), and three as borderline (8.8%; 95% CI: 3.0%-23.0%) (Table 1). Although ultrasound composition differed descriptively between malignant/borderline and benign lesions, the overall association was not statistically significant using the Fisher-Freeman-Halton exact test (p = 0.105; Cramér’s V = 0.57). The distributions of ultrasound composition, lesion margins, and calcification patterns according to histopathological diagnosis are summarized in Table 1.

**Table 1:** Histopathological classification of the study lesions

Histopathological category	Frequency, n/N (%)	95% CI
Malignant	16/34 (47.1%)	31.5%-63.3%
Benign	15/34 (44.1%)	28.9%-60.5%
Borderline	3/34 (8.8%)	3.0%-23.0%
Total	34/34 (100%)	-

The corresponding histopathological distribution is illustrated in Figure 1.

**Figure 1:**
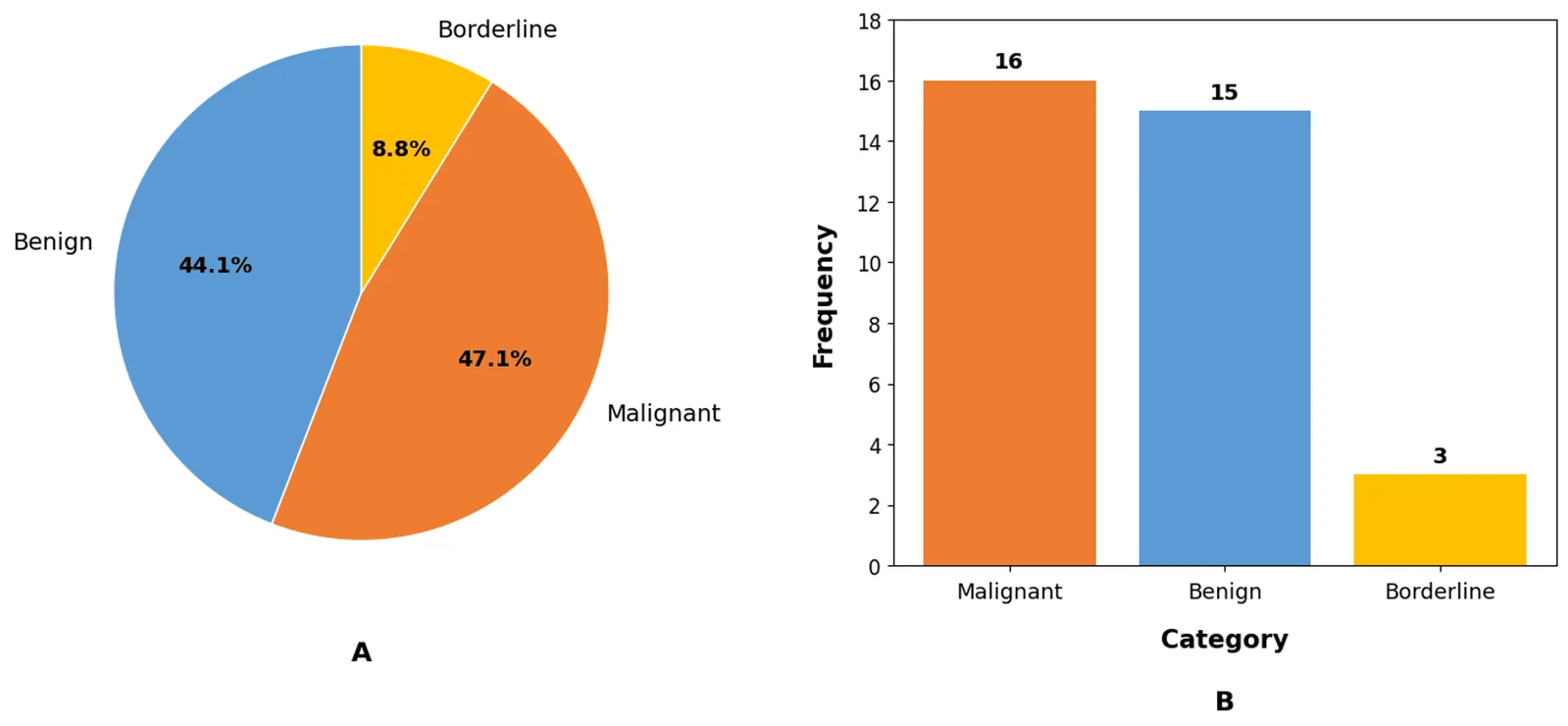
Histopathological distribution of the 34 adnexal-ovarian masses (A) Percentage distribution with corresponding counts and 95% confidence intervals: malignant, 16/34 (47.1%; 95% CI: 31.5% 63.3%); benign, 15/34 (44.1%; 95% CI: 28.9%-60.5%); and borderline, 3/34 (8.8%; 95% CI: 3.0%-23.0%). (B) Corresponding frequency distribution of malignant, benign, and borderline lesions.

Ultrasound composition

The lesions demonstrated variation in ultrasound composition across the malignant/borderline and benign histopathological groups. Solid lesions, papillary projections, and mural nodules were more frequently observed among malignant/borderline lesions, whereas predominantly cystic lesions were more commonly benign. However, the association between ultrasound composition and histopathological classification was not statistically significant (p = 0.144). The distributions of ultrasound composition, lesion margins, and calcification patterns according to histopathological diagnosis are summarized in Table 2.

**Table 2:** Ultrasound features and histopathological diagnosis

Ultrasound characteristic	Malignant/borderline, n/N (%)	Benign/non-malignant, n/N (%)	Total, n	p-value
Composition				0.105
Both solid and cystic	3/5 (60.0%)	2/5 (40.0%)	5	-
Predominantly cystic	0/2 (0.0%)	2/2 (100.0%)	2	-
Cystic	9/18 (50.0%)	9/18 (50.0%)	18	-
Solid	4/4 (100.0%)	0/4 (0.0%)	4	-
Solid with cystic components	0/2 (0.0%)	2/2 (100.0%)	2	-
Cystic with papillary projections	1/1 (100.0%)	0/1 (0.0%)	1	-
Cystic lesion with a smooth mural nodule	1/1 (100.0%)	0/1 (0.0%)	1	-
Papillary projections	1/1 (100.0%)	0/1 (0.0%)	1	-
Total	19/34 (55.9%)	15/34 (44.1%)	34	-
Margins				0.073
Irregular	5/15 (33.3%)	10/15 (66.7%)	15	-
Regular	8/11 (72.7%)	3/11 (27.3%)	11	-
Smooth	6/8 (75.0%)	2/8 (25.0%)	8	-
Total	19/34 (55.9%)	15/34 (44.1%)	34	-
Calcifications				0.247
Microcalcifications	5/7 (71.4%)	2/7 (28.6%)	7	-
Macrocalcifications	1/5 (20.0%)	4/5 (80.0%)	5	-
None	13/22 (59.1%)	9/22 (40.9%)	22	-
Total	19/34 (55.9%)	15/34 (44.1%)	34	-

The distribution of lesion composition across histopathological groups is depicted in Figure 2.

**Figure 2:**
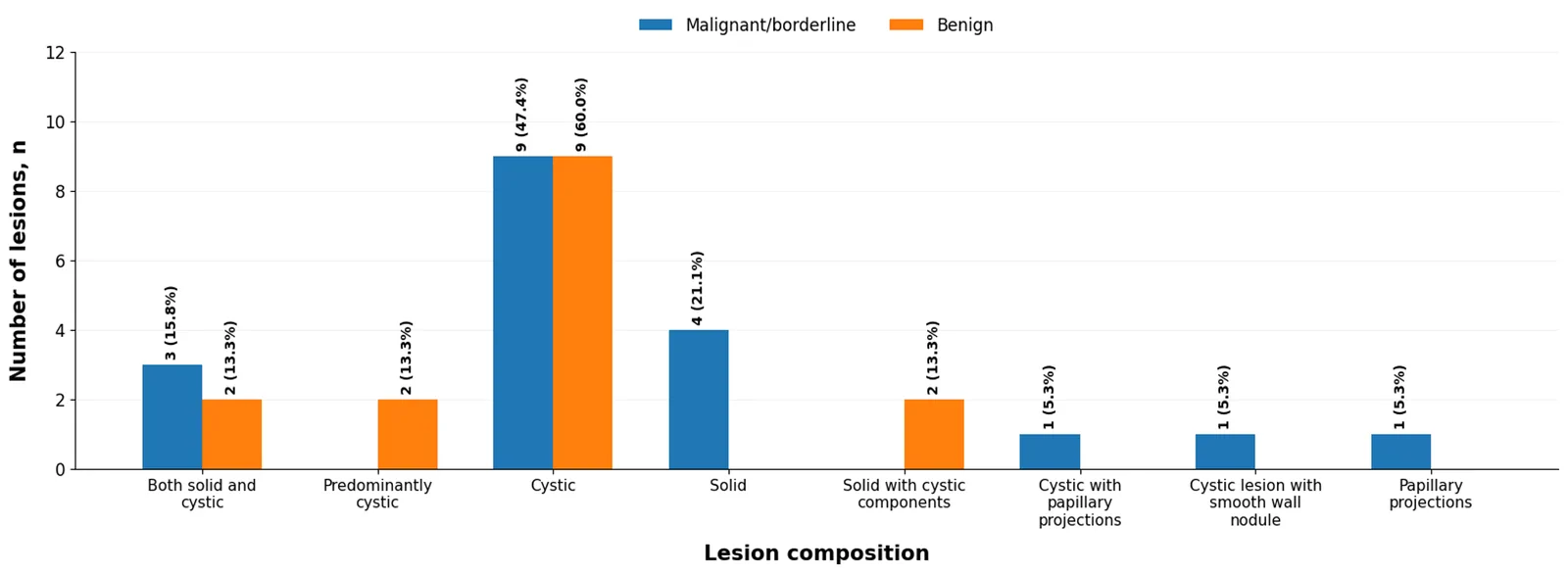
Distribution of lesion composition according to histopathological diagnosis Data labels are presented as n/N (%).

Lesion margins

Although lesion-margin patterns differed descriptively across the histopathological groups, the association between lesion margins and histopathological classification was not statistically significant (Fisher-Freeman-Halton exact p = 0.073; Cramér’s V = 0.40). The detailed distribution is shown in Figure 3.

**Figure 3:**
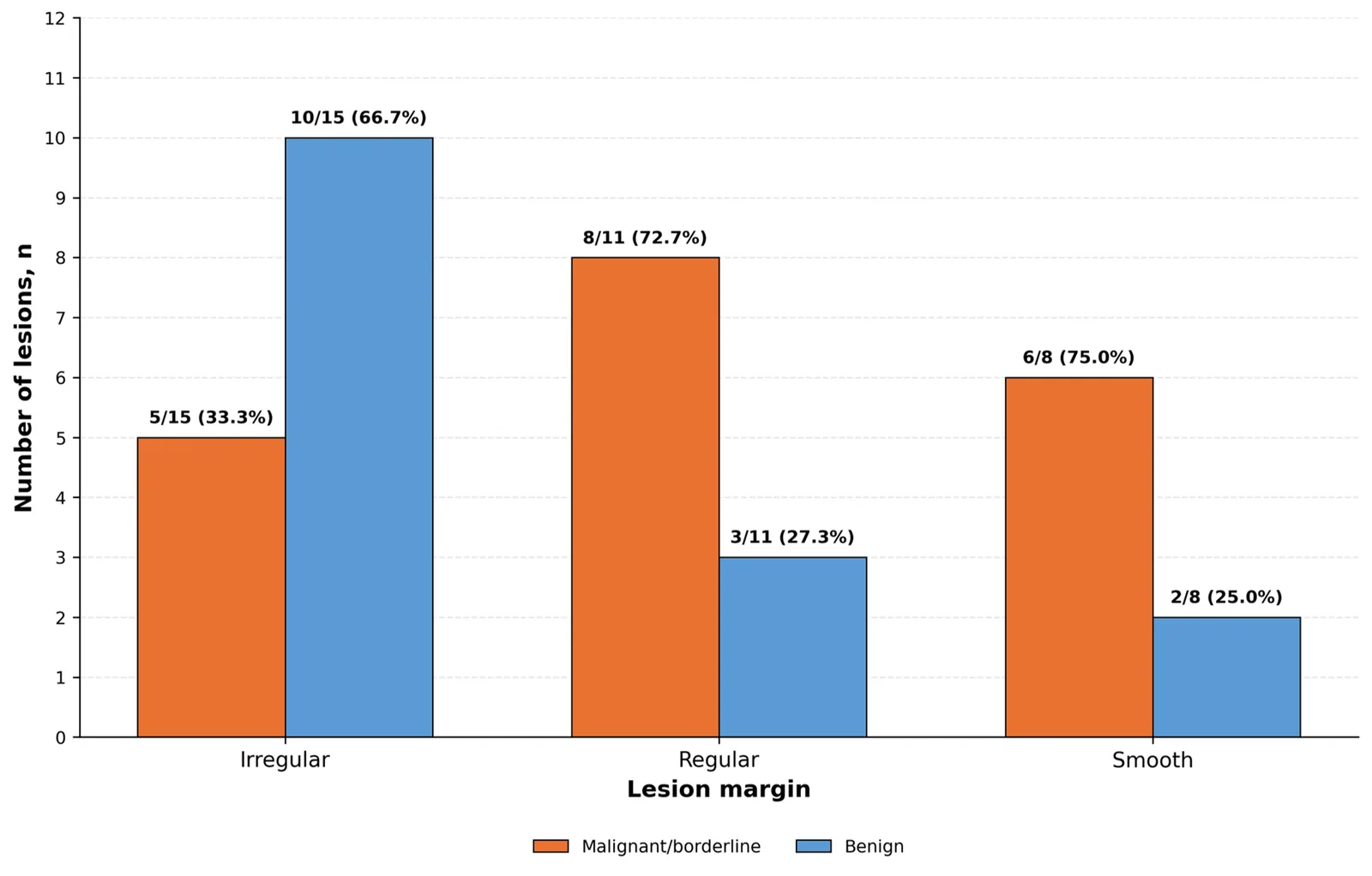
Histopathological distribution of adnexal-ovarian masses according to lesion-margin characteristics Values are presented as n/N (%).

Calcifications

Microcalcifications were more frequently observed among malignant/borderline lesions, whereas macrocalcifications were more frequently observed among benign lesions; however, the overall association between calcification pattern and histopathological classification was not statistically significant (Fisher-Freeman-Halton exact p = 0.247; Cramér’s V = 0.32). Distribution of calcification in Figure 4. 

**Figure 4:**
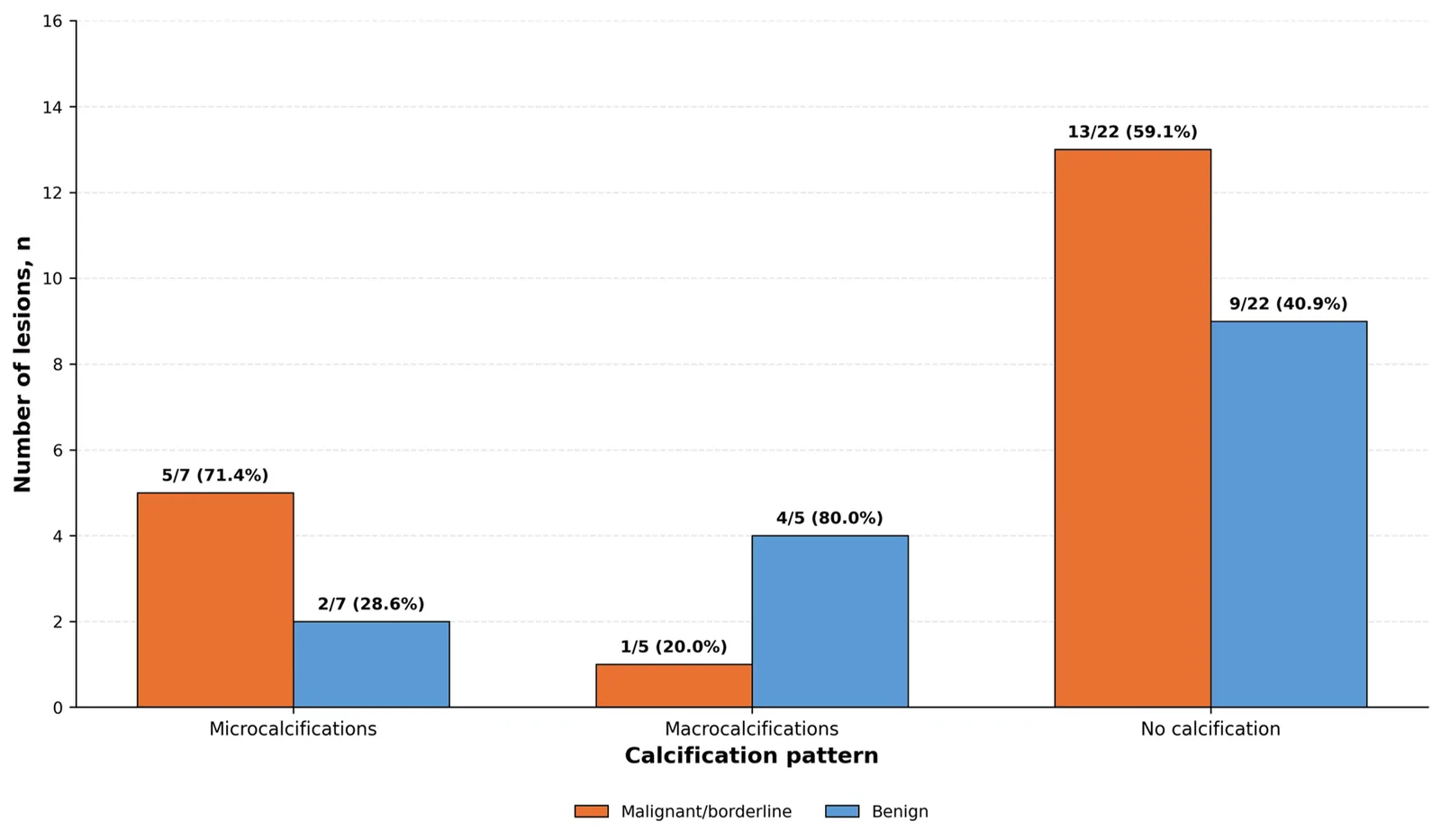
Distribution of calcification patterns according to histopathological diagnosis Data labels are presented as n/N (%).

Lesion locularity

Although ultrasound composition differed descriptively between malignant/borderline and benign lesions, the overall association was not statistically significant using the Fisher-Freeman-Halton exact test (p = 0.105; Cramér’s V = 0.57). The distributions of ultrasound composition, lesion margins, and calcification patterns according to histopathological diagnosis are summarized in Table 3.

**Table 3:** Association between lesion locularity and histopathological diagnosis Fisher-Freeman-Halton exact p = 0.075; Cramér’s V = 0.35.

Locularity	Malignant, n/N (%)	Benign, n/N (%)	Borderline, n/N (%)	Total, n
Multilocular	13/21 (61.9%)	7/21 (33.3%)	1/21 (4.8%)	21
Unilocular	2/11 (18.2%)	7/11 (63.6%)	2/11 (18.2%)	11
Unilocular predominantly solid	1/1 (100.0%)	0/1 (0.0%)	0/1 (0.0%)	1
Unilocular predominantly cystic	0/1 (0.0%)	1/1 (100.0%)	0/1 (0.0%)	1
Total	16/34 (47.1%)	15/34 (44.1%)	3/34 (8.8%)	34

The distribution of lesion locularity across the histopathological groups is shown in Figure 5.

**Figure 5:**
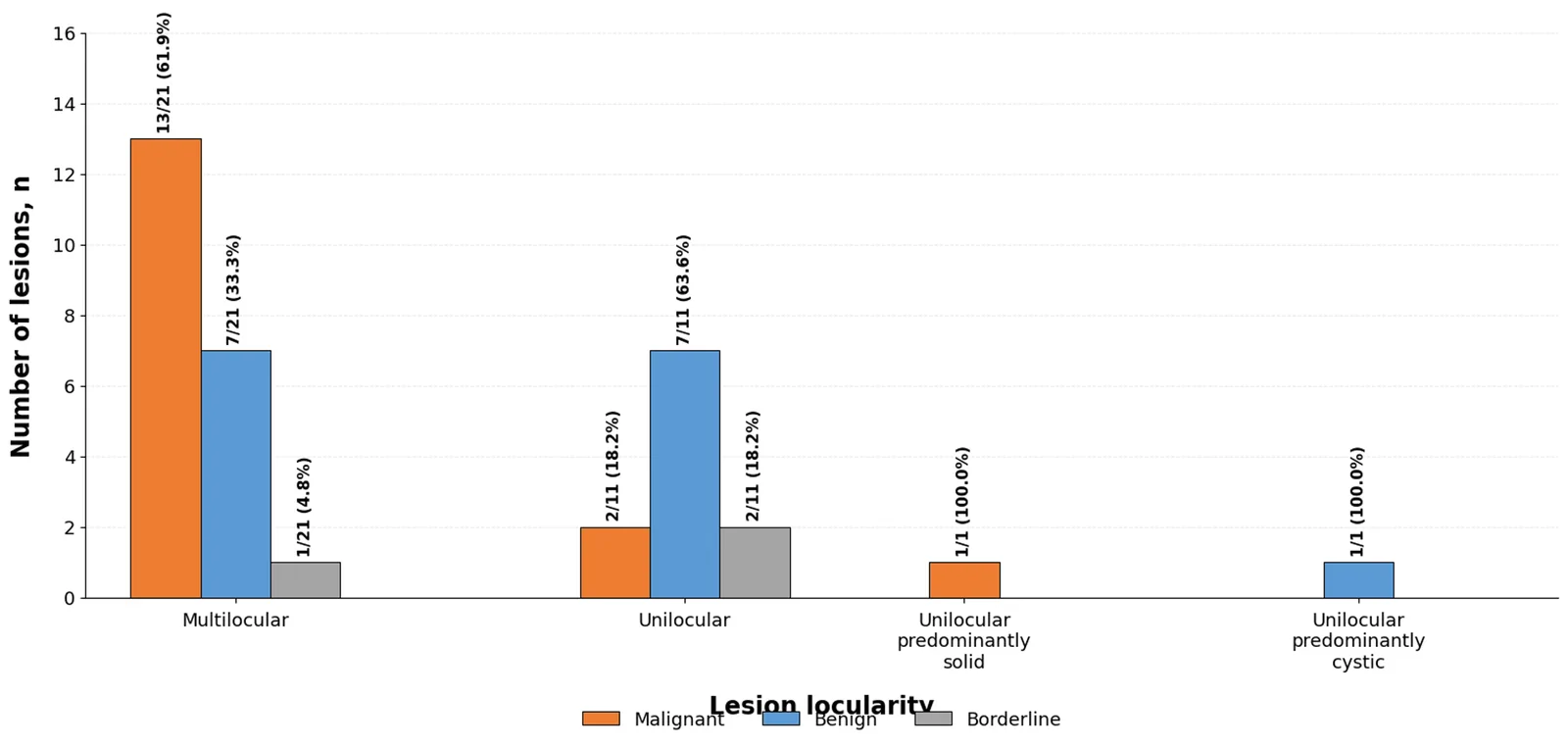
Distribution of lesion locularity according to histopathological diagnosis Data labels are presented as n/N (%).

Doppler vascularity

Doppler vascularity was additionally recorded using the 0-5 vascularity scoring scale documented in the original study dataset. Because the thesis does not provide categorical definitions for each score and this scale differs from the standard ACR O-RADS color-score terminology, these values were treated as study-recorded Doppler vascularity scores and analyzed descriptively rather than interpreted as the standardized ACR O-RADS color score. Doppler vascularity score showed a statistically significant association with binary histopathological classification (Fisher-Freeman-Halton exact p < 0.001; Cramér’s V = 0.81). The distribution of Doppler Color Score is presented in Table 4. 

**Table 4:** Doppler color score distribution

Colour score (CS)	Number of cases, n	Cases, n/N (%)
CS = 0	1	1/34 (2.9%)
CS = 1	6	6/34 (17.6%)
CS = 2	4	4/34 (11.8%)
CS = 3	12	12/34 (35.3%)
CS = 4	8	8/34 (23.5%)
CS = 5	3	3/34 (8.8%)
Total	34	34/34 (100.0%)

The association between Doppler vascularity score and histopathological classification is detailed in Table 5.

**Table 5:** Association between Doppler vascularity score and histopathological classification

Color score (CS)	Malignant/borderline, n/N (%)	Benign, n/N (%)	Total, n
CS = 0	0/1 (0.0%)	1/1 (100.0%)	1
CS = 1	0/6 (0.0%)	6/6 (100.0%)	6
CS = 2	0/4 (0.0%)	4/4 (100.0%)	4
CS = 3	9/12 (75.0%)	3/12 (25.0%)	12
CS = 4	8/8 (100.0%)	0/8 (0.0%)	8
CS = 5	2/3 (66.7%)	1/3 (33.3%)	3
Total	19/34 (55.9%)	15/34 (44.1%)	34

Association between O-RADS category and histopathological classification

All 10 O-RADS 3 lesions were benign. O-RADS 4 included 10/15 (66.7%) malignant, 4/15 (26.7%) benign, and 1/15 (6.7%) borderline lesions, whereas O-RADS 5 included 6/9 (66.7%) malignant, 1/9 (11.1%) benign, and 2/9 (22.2%) borderline lesions. The proportion of malignant lesions was identical in O-RADS 4 and O-RADS 5 (66.7% each). Although the combined proportion of malignant/borderline lesions was higher in O-RADS 5 than in O-RADS 4 (88.9% vs. 73.3%), the small sample did not demonstrate a clear stepwise increase in malignant proportion between these two categories. O-RADS category showed a significant overall association with histopathological classification using the Fisher-Freeman-Halton exact test (p < 0.001; Cramér’s V = 0.54), as summarized in Table 6.

**Table 6:** Association between O-RADS category and histopathological classification Values are presented as n/N (%) using row-wise percentages. The Fisher-Freeman-Halton exact test demonstrated a significant overall association between O-RADS category and three-category histopathological classification (exact p < 0.001; Cramér’s V = 0.54). O-RADS, Ovarian-Adnexal Reporting and Data System

O-RADS category	Malignant, n/N (%)	Benign, n/N (%)	Borderline, n/N (%)	Total, n
O-RADS 3	0/10 (0.0%)	10/10 (100.0%)	0/10 (0.0%)	10
O-RADS 4	10/15 (66.7%)	4/15 (26.7%)	1/15 (6.7%)	15
O-RADS 5	6/9 (66.7%)	1/9 (11.1%)	2/9 (22.2%)	9
Total	16/34 (47.1%)	15/34 (44.1%)	3/34 (8.8%)	34

The association between O-RADS category and binary histopathological classification is summarized in Table 7.

**Table 7:** Association between O-RADS category and binary histopathological classification Malignant and borderline lesions were combined as disease-positive for the binary analysis. The Fisher-Freeman-Halton exact test demonstrated a significant overall association between O-RADS category and binary histopathological classification (exact p < 0.001; Cramér’s V = 0.74). O-RADS, Ovarian-Adnexal Reporting and Data System

O-RADS category	Malignant/borderline, n/N (%)	Benign, n/N (%)	Total, n
O-RADS 3	0/10 (0.0%)	10/10 (100.0%)	10
O-RADS 4	11/15 (73.3%)	4/15 (26.7%)	15
O-RADS 5	8/9 (88.9%)	1/9 (11.1%)	9
Total	19/34 (55.9%)	15/34 (44.1%)	34

Diagnostic performance of O-RADS

For the binary diagnostic-accuracy analysis, the 16 malignant and three borderline lesions were combined as the disease-positive group, while the 15 benign lesions constituted the disease-negative group. O-RADS categories 4 and 5 were considered test-positive, whereas O-RADS category 3 was considered test-negative. All 19 malignant/borderline lesions were classified as O-RADS 4 or 5, resulting in 19 true-positive and no false-negative findings. Among the 15 benign lesions, 10 were classified as O-RADS 3 and five as O-RADS 4 or 5, resulting in 10 true-negative and five false-positive findings. O-RADS demonstrated a sensitivity of 100.0% (95% CI: 83.2%-100.0%), specificity of 66.7% (95% CI: 41.7%-84.8%), PPV of 79.2% (95% CI: 59.5%-90.8%), and NPV of 100.0% (95% CI: 72.2%-100.0%) (Table 8).

**Table 8:** Diagnostic performance of O-RADS using histopathological examination as the reference standard Malignant and borderline lesions were combined as disease-positive, whereas benign lesions were classified as disease-negative. O-RADS categories 4 and 5 were considered test-positive, and O-RADS category 3 was considered test-negative. Confidence intervals were calculated using the Wilson score method. O-RADS, Ovarian-Adnexal Reporting and Data System

Diagnostic measure	Calculation	Estimate, %	95% CI
Sensitivity	19/19	100.0	83.2-100.0
Specificity	10/15	66.7	41.7-84.8
Positive predictive value	19/24	79.2	59.5-90.8
Negative predictive value	10/10	100.0	72.2-100.0

The ROC analysis used the same reference-standard definition as the primary diagnostic-accuracy analysis, with malignant and borderline lesions classified as disease-positive and benign lesions classified as disease-negative. Using the ordinal O-RADS category as the test variable, the AUC was 0.870 (95% CI: 0.750-0.990; p < 0.001), indicating good discrimination between malignant/borderline and benign adnexal-ovarian masses in Figure 6.

**Figure 6:**
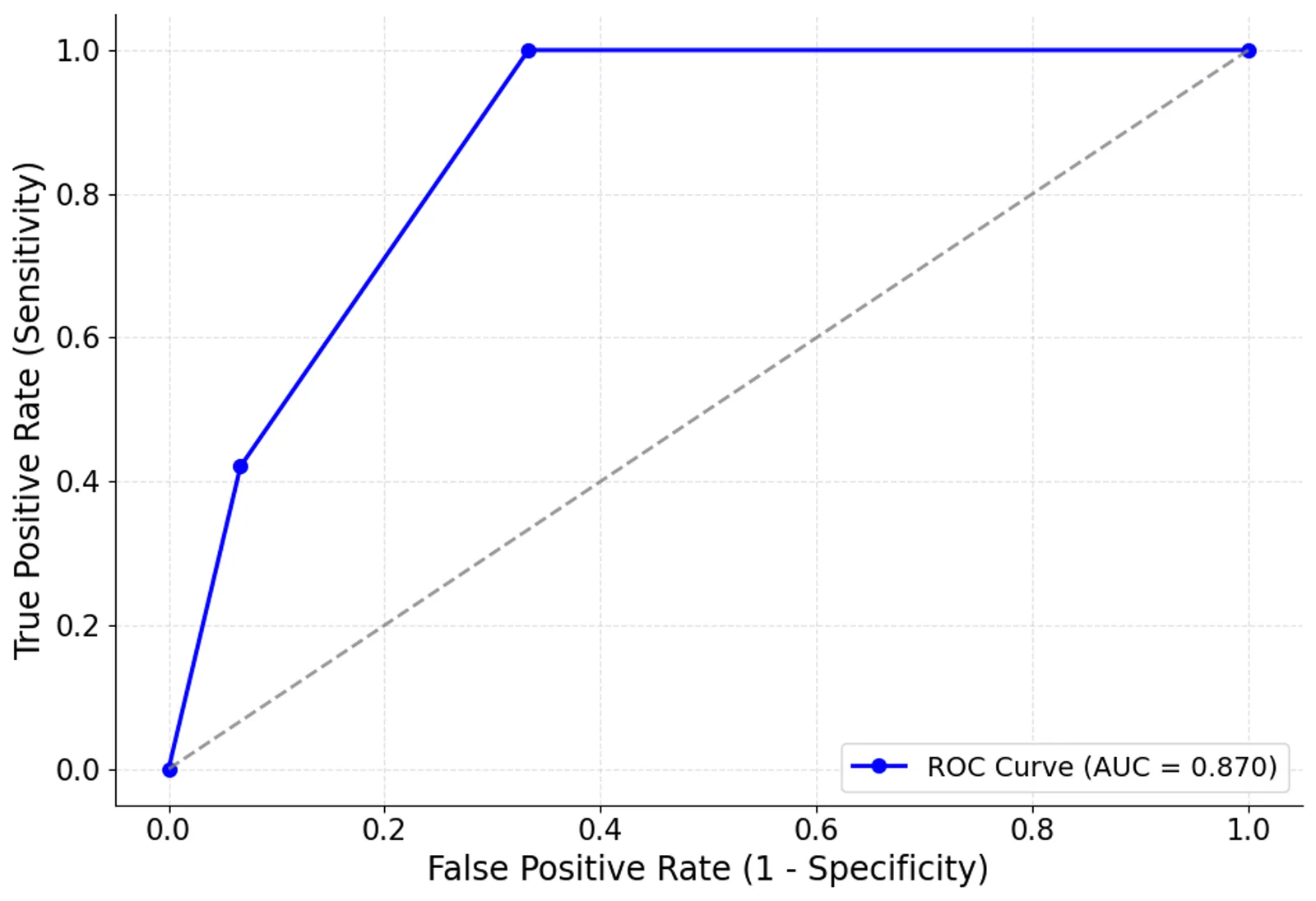
Receiver operating characteristic curve showing the diagnostic performance of O-RADS for distinguishing malignant/borderline from benign adnexal-ovarian masses (AUC: 0.870; 95% CI: 0.750-0.990) AUC, area under the receiver operating characteristic curve; O-RADS, Ovarian-Adnexal Reporting and Data System

## Discussion

Among the 34 lesions, 16/34 (47.1%) were malignant, 15/34 (44.1%) were benign, and 3/34 (8.8%) were borderline on histopathological examination. When O-RADS categories 4 and 5 were considered test-positive, all 19 malignant/borderline lesions were identified, yielding a sensitivity of 100.0% (95% CI: 83.2%-100.0%), specificity of 66.7% (95% CI: 41.7%-84.8%), PPV of 79.2% (95% CI: 59.5%-90.8%), and NPV of 100.0% (95% CI: 72.2%-100.0%). ROC analysis using the same disease-positive definition yielded an AUC of 0.870 (95% CI: 0.750-0.990). The high sensitivity and NPV should be interpreted cautiously because of the small sample size and corresponding wide CIs. All O-RADS 3 lesions were benign, whereas O-RADS 4 and 5 contained higher proportions of malignant/borderline lesions. Although several morphological features differed descriptively between groups, ultrasound composition (exact p = 0.105; Cramér’s V = 0.57), lesion margins (exact p = 0.073; Cramér’s V = 0.40), calcification pattern (exact p = 0.247; Cramér’s V = 0.32), and locularity (exact p = 0.075; Cramér’s V = 0.35) were not statistically significantly associated with histopathological classification. In contrast, Doppler vascularity showed a significant association with binary histopathological classification (exact p < 0.001; Cramér’s V = 0.81). O-RADS 4 and O-RADS 5 each contained 66.7% malignant lesions; although the combined malignant/borderline proportion was higher in O-RADS 5 than in O-RADS 4 (88.9% vs. 73.3%), the small number of lesions precluded interpretation of a clear stepwise malignancy gradient. Overall, the findings support an association between O-RADS category and histopathological outcome but should be interpreted cautiously because of the small sample and sparse cell distributions.

The observed diagnostic-performance estimates support the ability of ACR O-RADS to distinguish malignant/borderline from benign adnexal-ovarian masses within this cohort. O-RADS categories 4 and 5 identified all 19 malignant/borderline lesions, resulting in a sensitivity of 100.0% and an NPV of 100.0%; however, the corresponding CIs were wide, reflecting the small sample size. Specificity was 66.7%, indicating that some benign lesions were classified in higher-risk O-RADS categories. Therefore, the findings demonstrate promising diagnostic performance in this surgically selected, single-center cohort but do not establish that O-RADS can independently reduce unnecessary surgery or improve clinical outcomes. The results should be interpreted in the context of the limited sample size, surgical selection of cases, incomplete representation of O-RADS categories, and the need for external validation in larger populations. ROC analysis using the same disease-positive definition yielded an AUC of 0.870 (95% CI: 0.750-0.990), further supporting the discriminatory ability of O-RADS for separating malignant/borderline lesions from benign lesions in this cohort.

The diagnostic performance observed in the present study was broadly comparable with previous O-RADS validation studies, although specificity was lower in this small cohort. Cao et al. [10] reported a sensitivity of 98.7%, specificity of 83.2%, and AUC of 0.960 for O-RADS US in 1,054 adnexal masses. Another validation study reported an AUC of 0.941, sensitivity of 93.3%, and specificity of 90.2%. A more recent study evaluating O-RADS v2022 reported an AUC of 0.970, indicating higher discriminatory performance than observed in the present cohort. The higher sensitivity and NPV but lower specificity observed in the present study may partly reflect the small sample size, the relatively high proportion of malignant/borderline lesions, and differences in case selection and O-RADS category distribution. The results are consistent with the evidence that O-RADS ultrasound is diagnostic in various clinical situations. A two-center study showed that O-RADS can make good discrimination between benign and malignant ovarian masses [11]. Comparative studies with expert radiologists and explainable deep-learning models also revealed that standardized O-RADS features still have clinical relevance when integrated into powerful diagnostic systems [12]. Overall, the combination of O-RADS and Cancer Antigen 125 has been shown to enhance the specificity, PPV, accuracy, and discriminatory performance over the use of O-RADS alone [13]. Both classifications were found to be useful in this model of adneXa, with the diagnostic value of each method being dependent on the type of lesion and the study population [14], as confirmed by direct comparison to the International Ovarian Tumor Analysis assessment of different neoplasias.

O-RADS MRI has also demonstrated good test-retest accuracy and interobserver agreement and can aid machine-learning applications [15]. Standardized O-RADS classification could enhance the diagnostic accuracy of both junior and senior sonologists and minimize variations due to operator experience [16]. O-RADS for MRI can be helpful for the characterization of sonographically indeterminate lesions and for allowing for more structured clinical decision-making [17]. Clinical validation of ultrasound-based O-RADS has shown to be clinically useful for indeterminate adnexal masses [18]. The 2022 update updated the names of the ultrasound findings, risk category, and management recommendations to make the terminology more specific and more applicable [19]. Staging suspected malignancy is also recommended with MRI, which is supported by cross-sectional imaging recommendations [20] and computed tomography. The system's intent to stratify the risk based on the O-RADS has been observed in the distribution of benign, malignant, and borderline findings in O-RADS 4 and 5. These characteristics are associated with malignancy and should be evaluated together and not as individual features.

O-RADS may help standardize ultrasound reporting and facilitate communication between radiologists and gynecologists. Clinical management decisions should continue to integrate imaging findings with the broader clinical context. For the primary binary diagnostic-accuracy analysis, borderline and malignant lesions were combined as disease-positive because the analysis was designed to evaluate the ability of O-RADS to distinguish non-benign pathology from benign lesions using histopathology as the reference standard. Borderline tumors were nevertheless retained as a separate histopathological category in the descriptive analyses because they represent a distinct pathological entity.

Limitations and future directions

This study has several strengths, including its prospective diagnostic-accuracy design, use of histopathological examination as the reference standard, application of a standardized ACR O-RADS ultrasound framework, and reporting of diagnostic-performance estimates with 95% CIs. Several limitations should also be acknowledged. The study was conducted at a single center and included only 34 surgically evaluated adnexal-ovarian masses, including three borderline tumors, which limits statistical precision and generalizability. Because histopathological verification was available only for surgically excised lesions, selection and verification bias may have occurred, and the study population may not represent the full spectrum of adnexal masses encountered in routine clinical practice. Not all O-RADS categories were represented, limiting category-specific interpretation. Menopausal status was not systematically available for all participants. Formal blinding between ultrasound interpretation and histopathological assessment was not documented, and interobserver agreement was not evaluated. Information regarding transducer specifications, reader number and experience, the interval between ultrasonography and surgery, and the prespecified handling of bilateral or multiple lesions was also incompletely documented. Several contingency tables contained sparse cells and zero counts, increasing statistical uncertainty; exact statistical methods were therefore used where appropriate. The combination of borderline and malignant lesions into a single disease-positive group for the primary diagnostic-accuracy analysis may obscure differences in their biological behavior and imaging characteristics. This approach permitted a binary comparison with benign lesions; however, only three borderline tumors were included, precluding reliable assessment of O-RADS performance for borderline tumors separately.

Larger, consecutively recruited multicenter prospective studies with broader representation across O-RADS categories are needed to validate these findings. Future studies should use standardized imaging protocols, clearly documented blinding procedures, predefined approaches for bilateral or multiple lesions, and formal assessment of interobserver agreement. Evaluation of menopausal status, serum biomarkers, MRI, and emerging artificial intelligence-based approaches may further clarify their incremental value in the assessment of indeterminate adnexal masses.

## Conclusions

In this prospective cohort of 34 surgically evaluated adnexal-ovarian masses, ACR O-RADS ultrasound demonstrated promising diagnostic performance for distinguishing malignant/borderline from benign lesions. O-RADS categories 4 and 5 identified all malignant/borderline lesions in this cohort, resulting in high sensitivity and NPV; however, these estimates should be interpreted cautiously because of the small sample size, single-center design, wide CIs, and surgically selected study population. These findings require confirmation in larger, consecutively recruited multicenter populations before broader conclusions regarding clinical utility can be made.
